# Role of Long Noncoding RNAs in the Regulation of Cellular Immune Response and Inflammatory Diseases

**DOI:** 10.3390/cells11223642

**Published:** 2022-11-17

**Authors:** Fen Feng, Peng Jiao, Jinpeng Wang, Yanxia Li, Binwu Bao, Zhuoma Luoreng, Xingping Wang

**Affiliations:** 1 School of Agriculture, Ningxia University, Yinchuan 750021, China; 2 Key Laboratory of Ruminant Molecular Cell Breeding, Ningxia Hui Autonomous Region, Yinchuan 750021, China

**Keywords:** lncRNA, immunity, inflammatory diseases, gene expression, regulatory mechanism

## Abstract

Long noncoding RNAs (lncRNAs) are recently discovered genetic regulatory molecules that regulate immune responses and are closely associated with the occurrence and development of various diseases, including inflammation, in humans and animals. Under specific physiological conditions, lncRNA expression varies at the cell or tissue level, and lncRNAs can bind to specific miRNAs, target mRNAs, and target proteins to participate in certain processes, such as cell differentiation and inflammatory responses, via the corresponding signaling pathways. This review article summarizes the regulatory role of lncRNAs in macrophage polarization, dendritic cell differentiation, T cell differentiation, and endothelial and epithelial inflammation. In addition, it describes the molecular mechanism of lncRNAs in acute kidney injury, hepatitis, inflammatory injury of the lung, osteoarthritis, mastitis, and neuroinflammation to provide a reference for the molecular regulatory network as well as the genetic diagnosis and treatment of inflammatory diseases in humans and animals.

## 1. Introduction

Inflammation is a timely response of the body against tissue damage caused by pathogenic infection, chemical exposure, or physical damage, and it is a complex protective process that requires the interaction of different immune cells to eliminate or neutralize a harmful stimulus [1]. However, if the prolonged inflammatory response is not properly controlled, irreversible tissue damage, organ failure, or even death may occur. Long noncoding RNAs (lncRNAs) are newly discovered noncoding RNAs that are >200 nucleotides in length, have a limited ability to encode proteins, and have poor sequence conservation [2,3]. Many lncRNAs are key regulatory agents that participate in specific physiological and pathological processes via transcriptional or post-transcriptional regulatory mechanisms. LncRNAs participate in the regulation of several biological processes, including immune responses, inflammatory responses, cell proliferation, differentiation, apoptosis, and others [4]. In addition, lncRNAs play a key role in inflammation, primarily via the inflammatory signaling pathways that include JAK-STAT, MAPK, and NF-κB [5,6,7]. This article reviews the mechanisms of how lncRNAs are able to regulate the differentiation and inflammatory processes in different cell types. In particular, this review will focus on the molecular regulatory mechanisms of lncRNAs associated with the inflammation of the kidney, lung, liver, and central nervous system, as well as with diseases such as osteoarthritis (OA) and mastitis. Herein, we have sought to provide a reference for future in-depth studies on inflammatory diseases in humans and animals.

## 2. Function and Mechanism of lncRNAs

LncRNAs are synthesized via a pathway similar to that of protein-coding genes, with comparable histone modification profiles, splicing signals, and exon/intron lengths [8]. Most lncRNAs are transcribed by RNA polymerase II from genomic loci with chromatin states similar to those of mRNAs, and they are usually 5′-capped, spliced, and polyadenylated [9,10]. LncRNAs can be classified into sense lncRNAs, antisense lncRNAs, bidirectional lncRNAs, intergenic lncRNAs, and intragenic lncRNAs [11]. Although lncRNAs were originally considered the “dark matter” of the genome, with advances in molecular research, they are now recognized as molecules with distinct functional roles that regulate a range of cellular functions [10,12]. LncRNAs act as a decoy molecule of RNA, thereby interfering with the binding of transcription factors with the promoter region of target genes and inhibiting gene transcription. Furthermore, lncRNAs can recruit chromatin modifiers to regulate chromatin remodeling. Besides, lncRNAs can also play a role as competing endogenous RNA (ceRNA), i.e., they can compete with and regulate the expression of the target gene of miRNAs. In addition, lncRNAs can directly bind to mRNAs, causing their transcriptional inhibition, shear regulation, or direct degradation. Finally, lncRNAs act as a “bridge” between ribonucleoproteins and proteins to regulate gene expression [13,14]. To summarize, at the epigenetic level, lncRNAs regulate gene expression by modulating allele expression, chromatin modification, and genomic imprinting. At the transcriptional level, lncRNAs regulate gene expression by interacting with proteins or DNA. At the post-transcriptional level, they are involved in regulating mRNA translation, degradation, and alternative splicing [15].

## 3. Role of lncRNAs at the Cellular Level

LncRNAs participate in the proliferation and differentiation of macrophages, dendritic cells (DCs), and T cells as well as in the inflammatory response of endothelial cells and epithelial cells via different regulatory mechanisms. Different lncRNAs target their specific miRNAs, genes, or proteins to exert their effects (Table 1).

### 3.1. Role of lncRNAs in Macrophage Polarization

**Figure 1:**
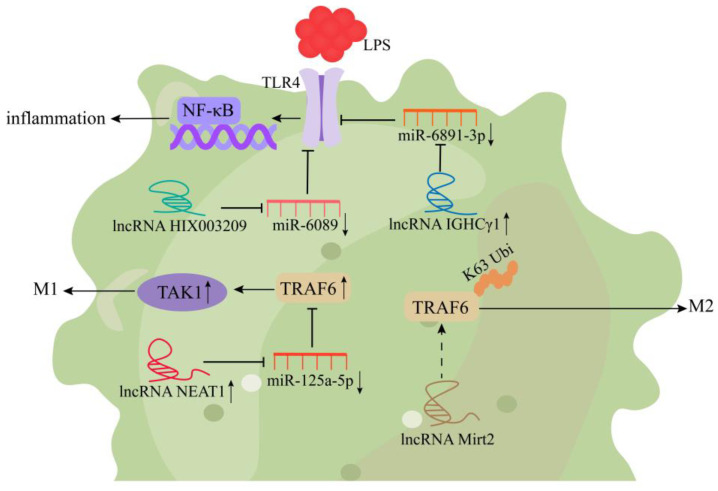
Mechanism of lncRNAs for regulating macrophage proliferation and differentiation. LncRNA Mirt2 promotes the M2 polarization of macrophages by inhibiting the Lys63 (K63) ubiquitination of TRAF6. LncRNA NEAT1 increases the expression of TRAF6 and TAK1 by binding to miR-125a-5p, eventually leading to M1 polarization of macrophages. Overexpression of lncRNA IGHCγ1 promotes the activation of the NF-κB signaling pathway by regulating miR-6891-3p/TLR4 axis, thereby aggravating the TLR4-mediated inflammatory response of macrophages. LncRNA HIX003209 promotes the expression of TLR4 and activation of NF-κB in macrophages by targeting miR-6089.

Macrophages, the key members of innate immunity, play vital roles in inflammatory responses, autoimmune responses against viral infections, and tumorigenesis. In addition, they participate in adaptive immune responses through antigen processing and presentation and provide downstream effector functions [46,47]. Of note, the functional transition of macrophages is closely associated with the pathogenesis of inflammatory diseases [48]. Research has shown that some lncRNAs play important roles in the activation and differentiation of macrophages and are involved in the regulation of macrophage polarization and inflammatory responses (Figure 1) [49]. In lipopolysaccharide (LPS)-induced mouse macrophages, tumor necrosis factor (TNF) receptor-associated factor 6 (TRAF6) assists TLR4 in mediating the activation of the downstream NF-κB signaling pathway. LncRNA Mirt2 alleviates the inflammatory response after TLR4 activation by inhibiting the Lys63 (K63) ubiquitination of TRAF6 and promotes the anti-inflammatory (M2) polarization of macrophages, which alleviates macrophage inflammation (Figure 1) [16]. Wang et al. [17] showed that the expression of lncRNA NEAT1 was significantly increased in LPS-induced mouse RAW264.7 macrophages. LncRNA NEAT1 increased the expression of TRAF6 and the phosphorylation of transforming growth factor-β-activated kinase 1 (TAK1) protein by binding to miR-125a-5p, eventually leading to LPS-induced macrophage polarization toward M1 and possibly ameliorating LPS-induced sepsis (Figure 1). LncRNA NEAT1 also upregulates the expression of NLRP3 in macrophages, thereby promoting the occurrence of inflammatory responses [18]. Moreover, the expression of lncRNA IGHCγ1 is significantly upregulated in macrophages, which promotes the expression of TLR4 by binding to miR-6891-3p, induces the activation of the NF-κB signaling pathway, and aggravates the TLR4-mediated inflammatory response of macrophages (Figure 1) [19]. Yan et al. [20] showed that lncRNA HIX003209 promotes the expression of TLR4 and activation of NF-κB in macrophages by targeting miR-6089. Therefore, knockdown of lncRNA HIX003209 helps to alleviate inflammation in macrophages and may be useful in developing a therapeutic strategy for rheumatoid arthritis (Figure 1).

The control of monocyte/macrophage differentiation is a complex process that requires a coordinated expression of stage-specific transcription factors, cytokines, and noncoding RNAs [50]. During monocyte/macrophage differentiation, PU.1 acts as a transcriptional repressor to negatively regulate the expression of miR-199a-5p and directly activates lnc-MC. The upregulation of lnc-MC absorbs miR-199a-5p for the further enhancement of the role of PU.1 during differentiation. Owing to this process, the inhibition of activin A receptor type 1B gene expression is alleviated, and the transforming growth factor-β (TGF-β) signaling pathway is activated, which promotes monocyte/macrophage differentiation [21,51]. Thus, lnc-MC acts as a ceRNA to regulate monocyte production. In summary, the balance between macrophage M1/M2 polarization has been identified as a key determinant of inflammatory disease development, and the enhancement of macrophage M2 polarization at an early stage may reasonably suppress the development of immune and inflammatory responses. Hence, lncRNAs regulate macrophage M1/M2 polarization homeostasis and function by binding to specific targets, improve immune responses, and prevent the development of inflammatory disease.

### 3.2. Regulatory Effects of lncRNAs on Dendritic Cells

DCs are typical antigen-presenting cells that play a vital role in linking the innate and adaptive immune processes and influence the pathological mechanisms of various immune diseases [52]. Owing to the unique role of DCs in immune diseases, researchers have recently increasingly studied the regulation of lncRNAs in the immune-related disease mechanisms and pathological processes associated with DCs.

**Figure 2:**
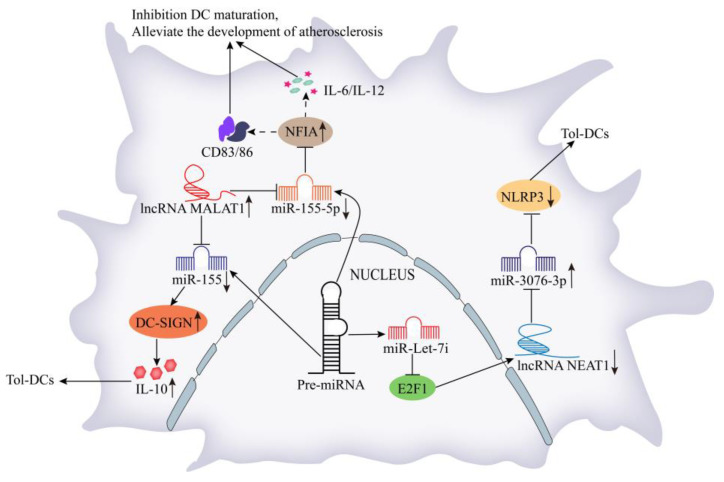
Mechanism of the regulatory role of lncRNAs in dendritic cells. MiR-let-7i regulates the expression of lncRNA NEAT1 by binding E2F1, and the downregulated lncRNA NEAT1 promotes the polarization of DCs to a more tolerant phenotype via miR-3076-3p/NLRP3 axis. LncRNA MALAT1 promotes DC-SIGN and IL-10 by targeting miR-155 in the cytoplasm of DCs, thereby regulating the formation of tolerogenic DCs. Overexpression of lncRNA MALAT1 inhibits DC maturation by regulating the miR-155-5p/NFIA axis.

LncRNAs participate in regulating the differentiation and function of DCs via different regulatory pathways. LncRNA Dpf3 is upregulated in chemokine receptor 7-induced DC migration. LncRNA Dpf3 binds to HIF-1α, thereby inhibiting DC migration and alleviating the inflammatory damage caused by their abnormal migration [22]. Zhang et al. [23] found that the expression of lncRNA NEAT1 was significantly increased during LPS-induced DC maturation, and miR-let-7i regulates the expression of lncRNA NEAT1 by binding to transcription factor E2F1. Meanwhile, lncRNA NEAT1 acts as a ceRNA to regulate NLRP3 expression by targeting miR-3076-3p. In experimental autoimmune myocarditis and heart transplantation mouse models, the knockout of lncRNA NEAT1 reduced the infiltration of inflammatory cells, increased the number of regulatory T (Treg) cells, and promoted the polarization of DCs to a more tolerant phenotype (Figure 2). Moreover, lncRNA MALAT1 was involved in the tolerogenic DC induction and immune tolerance regulation in heart transplantation and experimental autoimmune myocarditis models. LncRNA MALAT1 promotes the production of DC-specific intercellular adhesion molecule-3 grabbing nonintegrin (DC-SIGN) and interleukin (IL)-10 by targeting miR-155 in the cytoplasm of DCs, thereby regulating the formation of tolerogenic DCs and leading to immune tolerance (Figure 2) [24]. LncRNA MALAT1 is significantly downregulated in oxidized low-density lipoprotein (ox-LDL)-induced DCs. Overexpression of lncRNA MALAT1 suppresses the production of IL-6, IL-10, CD83, and CD86 by regulating the miR-155-5p/nuclear factor I/A (NFIA) axis, which inhibits ox-LDL-induced DC maturation and ultimately alleviates the development of atherosclerosis (Figure 2) [25].

Signal transducer and activator of transcription 3 (STAT3) is a transcription factor that regulates DC differentiation. Lnc-DC binds to STAT3 in the cytoplasm and promotes DC differentiation via the STAT3 signaling pathway, and knockdown of lnc-DC reduces the ability of human monocytes to differentiate into DCs [26]. In hepatitis B virus-induced DCs, knockdown of lnc-DC reduced the expression levels of pSTAT3, TLR9, and SOCS3 and promoted apoptosis in DCs [27]. Thus, lncRNAs play crucial roles in the migration, maturation, and differentiation of DCs. Different lncRNAs participate in regulating DC differentiation through various regulatory pathways, improving inflammatory damage in the body and preventing the occurrence of inflammation. However, limited studies have focused on the regulatory mechanisms of lncRNAs on DCs. Therefore, additional research on this topic may provide a more solid foundation for the diagnosis and treatment of immune diseases.

### 3.3. Role of lncRNAs in T Cell-Mediated Inflammation

T cells are the primary component of lymphocytes and perform various biological functions that help resist disease infection and tumor formation in the body. Mature T cells are distributed to the thymus-dependent area of peripheral immune organs through blood flow and can be recycled via the lymphatic vessels, peripheral blood, and tissue fluid, to exert their functions in cellular immunity and immune regulation. LncRNAs can regulate T cell differentiation and function via different modes of action. Ranzani et al. [28] showed that linc-MAF-4 promoted Th1 cell differentiation by inhibiting the Th2 cell-associated transcription factor MAF-4. In addition, lncRNA AW112010 could inhibit the expression of IL-10 by binding to KDM5A, thereby promoting T cell differentiation and inhibiting inflammation [29,53]. In inflammatory bowel disease, lncRNA IFNG-AS1 is selectively overexpressed at genomic loci in T cells, and overexpressed lncRNA IFNG-AS1 promotes the expression of Th1 inflammatory cytokines IFNG and IL-2, which increases the differentiation of Th1 cells [30]. Liu et al. [31] detected a significant downregulation of lncRNA GAS5 expression in CD4+ T cells from patients with systemic lupus erythematosus. The overexpression of lncRNA GAS5 could promote the level of E4 binding protein 4 (E4BP4) through the sponge adsorption of miR-92a-3p, ultimately inhibiting the activation of CD4+ T cells. Thus, lncRNAs are important regulators of T cell differentiation and are associated with inflammatory diseases by modulating T cell differentiation. These T cell-regulating lncRNAs can, therefore, be potential therapeutic targets.

### 3.4. Role of lncRNAs in Endothelial Cell Inflammation

Atherosclerosis results from a series of inflammatory responses due to vascular endothelial cell injury, and endothelial cell apoptosis disrupts the integrity of the vascular endothelium. Ox-LDL-induced endothelial cell dysfunction is an important factor in the mechanism of atherosclerosis development. Ox-LDL affects atherosclerosis via three pathways: endothelial cell injury, inflammatory response, and increased oxidative stress [54,55].

**Figure 3:**
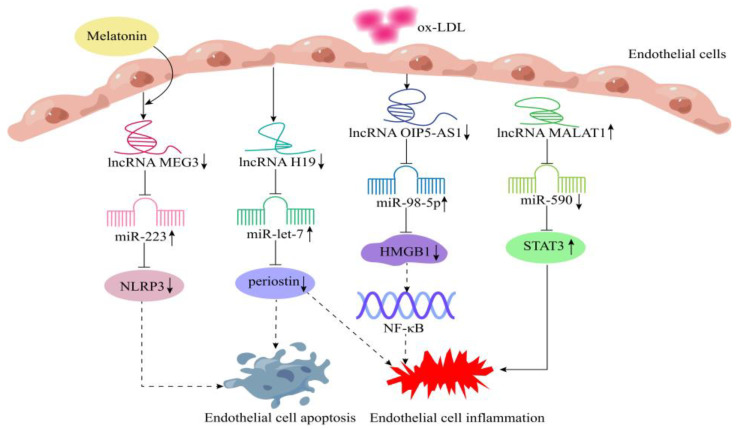
Mechanism of lncRNAs in endothelial cells inflammation. Downregulation of lncRNA H19 inhibits inflammation and apoptosis in HUVECs by targeting miR-let-7/periostin. Knockdown of lncRNA OIP5-AS1 alleviates endothelial cell inflammation via regulating miR-98-5p/TLR4/NF-κB pathway. LncRNA MALAT1 promotes the inflammatory response of endothelial cells via regulating the miR-590/STAT3 axis. Melatonin prevents endothelial cell apoptosis via lncRNA MEG3/miR-223/NLRP3 axis in atherosclerosis.

LncRNAs play a critical role in the regulation of endothelial cells and vascular inflammation [56,57,58], such as lncRNA H19, which was the first lncRNA discovered in 1990 [59]. Cao et al. [32] found that lncRNA H19 expression was significantly upregulated in ox-LDL-induced human umbilical vein endothelial cells (HUVECs). LncRNA H19 downregulation could reduce periostin expression levels by targeting miR-let-7, thereby inhibiting inflammation, apoptosis, and oxidative stress in HUVECs (Figure 3). Zheng et al. [33] found that the expression of lncRNA OIP5-AS1 was significantly increased in ox-LDL-induced HUVECs, and knockdown of lncRNA OIP5-AS1 alleviated endothelial cell apoptosis and inflammation via regulating the miR-98-5p/TLR4/NF-κB pathway. (Figure 3). In addition, lncRNA MALAT1 expression was significantly upregulated in the ox-LDL-induced endothelial cell inflammation model. LncRNA MALAT1 upregulated STAT3 expression by sponge-adsorbing miR-590, which promoted the inflammatory response of endothelial cells and reduces the migration ability of cells. These pathways may provide new diagnostic and therapeutic strategies for the atherosclerotic cerebrovascular diseases caused by ox-LDL (Figure 3) [34].

It has been shown that melatonin has substantial anti-inflammatory properties and plays a vital role in atherosclerosis [60,61]. Zhang et al. [35] showed that lncRNA MEG3 was significantly upregulated in the endothelial cells of ApoE−/− mice treated with a high-fat diet, ox-LDL-mimicking atherosclerotic human aortic endothelial cells in vitro. Melatonin reduces the expression of lncRNA MEG3, and the low expression of lncRNA MEG3 inhibits the apoptosis of aortic endothelial cells via regulation of the miR-223/NLRP3 axis, thereby alleviating atherosclerosis (Figure 3). This indicates that some drugs may be able to alleviate inflammatory damage in endothelial cells by regulating miR-223 expression through lncRNAs.

In angiotensin II (Ang II)-induced hypertensive mice and Ang II-induced HUVEC injury models, Qian et al. [36] found that the expression of lncRNA SNHG12 was significantly downregulated. The overexpression of lncRNA SNHG12 targeted miR-25-3p to increase the expression of sirtuin (SIRT) 6. This process alleviates the vascular endothelial injury induced by hypertension, providing a potential target for the treatment of Ang II-induced hypertension. In an in vitro uric acid-induced HUVECs injury model, lncRNA-HOTAIR regulated NLRP3 expression by competitively binding to miR-22, thereby promoting endothelial cell pyroptosis and inflammatory responses as well as causing renal injury [37]. Hence, elucidating the molecular mechanisms of lncRNA action in endothelial cells can help to better investigate the underlying mechanisms of endothelial cell-associated inflammatory diseases.

### 3.5. Role of lncRNAs in Epithelial Cell Inflammation

LncRNAs perform important functions in epithelial cells. For example, in high glucose-stimulated retinal pigment epithelial (ARPE-19) cells, the expression of lncRNA H19 was significantly reduced, and the overexpressed lncRNA H19 targeted miR-19b to increase the expression of SIRT1, thereby inhibiting the high glucose-induced inflammatory response in ARPE-19 cells [38]. In addition, lncRNA MEG3 expression was significantly downregulated in ARPE-19 under high glucose conditions, and the overexpression of lncRNA MEG3 promoted SIRT1 expression by downregulating miR-34a. This process inhibited high glucose-induced apoptosis and inflammatory factor secretion, indicating a novel idea for evaluating therapeutic measures for diabetic retinopathy [39].

In NPS-Nd2O3-treated human bronchial epithelial cells, lncRNA loc105377478 promoted NF-κB activation by negatively regulating the expression of AdipoR1, which upregulated IL-6 and IL-8 expression to promote inflammatory responses in human bronchial epithelial cells [40]. Ji et al. [41] revealed that the expression of lncRNA Hsp4 in LPS-induced alveolar epithelial cell MLE-12 was significantly reduced. The overexpressed lncRNA Hsp4 sponge-acted on miR-466m-3p to increase the expression of DNAJB6 and inhibit LPS-induced alveolar epithelial cell apoptosis, which may be a potential target for the diagnosis and treatment of acute lung injury (ALI). Jiang et al. [42] found that knockdown of lncRNA NEAT1 could reduce HIF-1α expression by binding and interacting with miR-582-5p, inhibiting PM2.5-induced epithelial–mesenchymal transition (EMT) in lung bronchial epithelial cells and preventing the acquisition of cancer stem cell-like properties. In addition, the expression of lncRNA NEAT1 was upregulated in the sera of patients with sepsis and in the LPS-induced human renal tubular epithelial cell line HK-2. LncRNA NEAT1 worsened LPS-induced HK-2 cell injury by acting as a sponge for miR-93-5p to regulate TXNIP expression [43]. Xu et al. [44] revealed that in the LPS-induced HK-2 cell inflammatory injury model, lncRNA TUG1 inhibited the NF-κB pathway by regulating the expression of miR-223 and SIRT1, which protected HK-2 cells from the LPS-induced inflammatory damage.

Of note, lncRNAs are involved in regulating the proliferation and differentiation of mammary epithelial cells. However, few studies have evaluated lncRNAs in the mammary epithelial cells of dairy cows, and functional studies are rather lacking [62,63]. In bovine mammary epithelial cells, lncRNA MPNCR competitively binds to miR-31 as a ceRNA, upregulates the expression of the miR-31 target gene CAMK2D, and subsequently inhibits the proliferation of bovine mammary epithelial cells [45]. Therefore, lncRNAs may be used as a new potential therapeutic target for epithelial cell inflammatory diseases. However, the function and mechanism of action of lncRNAs in bovine mammary epithelial cells still need further evaluation.

## 4. Role of lncRNAs in Inflammatory Diseases

LncRNAs regulate inflammatory factor expression and inflammatory signaling pathways by interacting with specific miRNAs, mRNAs, and proteins at the transcriptional and post-transcriptional levels, ultimately alleviating inflammatory damage. At present, some progress has been made regarding research on lncRNAs in the diagnosis and treatment of inflammatory diseases such as acute kidney injury (AKI) [64], liver inflammation [65], ALI [66], OA [67], mastitis [68], and neuroinflammation [69] (Table 2). This section primarily focuses on the roles of lncRNAs in inflammatory diseases, to provide some reference for further research on lncRNAs in inflammatory diseases.

### 4.1. Role of lncRNAs in AKI

**Figure 4:**
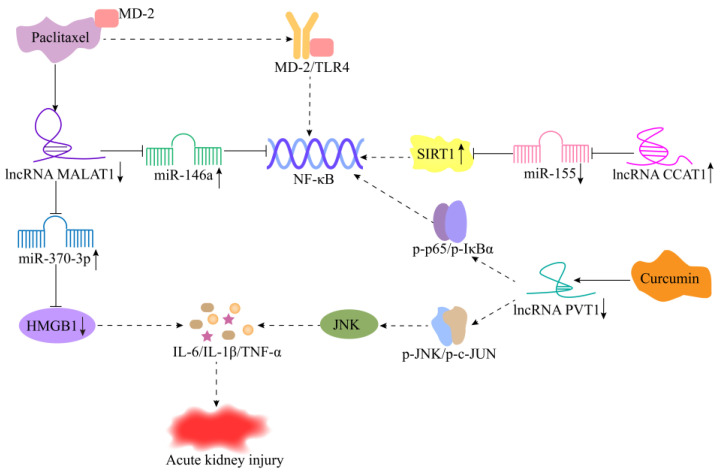
Mechanism of lncRNAs in acute renal injury. LncRNA CCAT1 inhibits the activation of NF-κB via regulating the miR-155/SIRT1 axis. Paclitaxel binds to MD-2 to block MD-2/TLR4 association, resulting in the suppression of NF-κB activation. In addition, paclitaxel can protect against AKI via the regulation of lncRNA MALAT1/miR-370-3p/HMGB1 axis. Knockdown of lncRNA MALAT1 inhibits the activation of NF-κB by targeting miR-146a. Curcumin attenuates the activation of the JNK/NF-κB signaling pathway by inhibiting lncRNA PVT1, thereby alleviating the inflammatory response in AKI.

AKI, one of the most common renal inflammatory diseases, is a syndrome that requires intensive care. Its typical symptom is renal function injury, and the main causes include sepsis, hypoxia, trauma, and LPS induction. It is characterized by a rapid decline of renal function, which is also the reason for the high incidence rate and patient mortality in intensive care units [111]. At present, compared with the widely reported differentially expressed miRNAs associated with various diseases, few functional studies on lncRNAs in renal cancer have been conducted. In addition, very few lncRNAs are associated with AKI development. LncRNA CCAT1, one of the first lncRNAs, plays roles in the pathogenesis of a disease and has a protective effect on AKI. The expression of lncRNA CCAT1 is decreased in a LPS-induced human renal tubular epithelial cell inflammation model. LncRNA CCAT1 prevents the LPS-induced renal cells’ apoptosis and inflammatory injury via regulation of the miR-155/SIRT1 axis. (Figure 4) [70]. Wang et al. [71] showed for the first time that lncRNA CRNDE inhibited the development of sepsis-induced AKI. The study revealed that lncRNA CRNDE expression was significantly downregulated in LPS-induced AKI in rats with sepsis and that lncRNA CRNDE upregulated peroxisome proliferator-activated receptor-α (PPAR-α) expression by targeting miR-181a-5p, which inhibited renal cell apoptosis and inflammatory injury.

Paclitaxel is currently the most widely used antitumor drug for treating various cancers. In recent years, studies have found that paclitaxel may play an anti-inflammatory role, and it has been confirmed as a potential therapeutic drug, in particular, for AKI [112,113]. In LPS-induced AKI in mice, paclitaxel may bind to MD-2 to block the MD-2/TLR4 association, resulting in the suppression of NF-κB activation and inhibition of proinflammatory cytokine production (Figure 4) [114]. LncRNA MALAT1 was significantly decreased in LPS-induced HK-2 cells under paclitaxel treatment. LncRNA MALAT1 targeted by miR-370-3p, thereby inhibiting the expression level of HMGB1 as well as the production of inflammatory factors and, subsequently, alleviating the inflammatory injury of AKI (Figure 4) [72]. Ding et al. [73] revealed that lncRNA MALAT1 regulates the NF-κB signaling pathway in LPS-induced AKI via the modulation of miR-146a expression, providing new insights into the complex molecular mechanisms of specific miRNAs and lncRNAs in LPS-induced AKI (Figure 4). In addition, the expression of lncRNA PVT1 was significantly elevated in the cells and tissues of LPS-induced AKI in sepsis. Curcumin attenuated the activation of the JNK/NF-κB signaling pathway by inhibiting lncRNA PVT1, thereby alleviating the inflammatory response in AKI in sepsis (Figure 4) [74,115].

Although the pathogenic factors of AKI are well-known, its complex biological and molecular mechanisms need detailed study via clinical and basic research. An in-depth study of the regulatory mechanism of lncRNA in AKI may be helpful in developing new therapeutic strategies in the future.

### 4.2. Role of lncRNAs in Hepatic Inflammatory Diseases

Hepatitis is the general term of liver inflammation, including acute liver injury, alcoholic liver disease, and liver fibrosis. It is usually caused by various pathogenic factors, including viruses, bacteria, alcohol, drugs, etc. [116]. It has been recently shown that lncRNAs are involved in the regulation of hepatic inflammatory diseases [117,118].

The LPS-induced cell or tissue inflammation model is a common model for studying lncRNAs in regulating hepatic inflammatory diseases. The expression of lncRNA HOTAIR was significantly upregulated in LPS-induced hepatocytes. Overexpressed lncRNA HOTAIR activated the NF-κB signaling pathway and promoted the expression of IL-1β, IL-6, and TNF-α, which activated the JAK2/STAT3 pathway and ultimately worsened LPS-induced inflammatory injury in hepatocytes [75]. Shen et al. [76] found that lncRNA XIST expression was significantly upregulated in the liver tissue of rats with sepsis-induced acute liver injury. In addition, they found that lncRNA XIST could directly bind to BRD4, and knockdown of lncRNA XIST significantly inhibited BRD4 expression and alleviated inflammatory injury. Liu et al. [77] revealed that lncRNA TUG1 was significantly highly expressed in LPS-induced mouse liver and silencing lncRNA TUG1 reduced TNF expression by targeting miR-140, which alleviated LPS-induced hepatocyte inflammation and injury.

**Figure 5:**
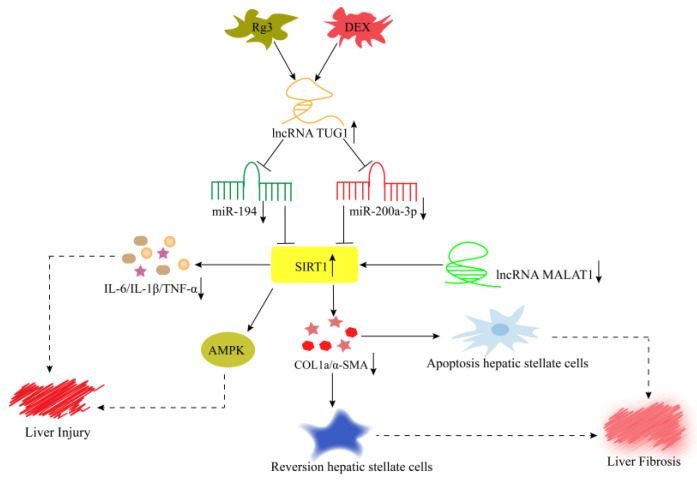
Mechanism of lncRNAs in hepatic inflammatory diseases. DEX suppresses the inflammatory response of hepatocytes by mediating of lncRNA TUG1/miR-194/SIRT1 axis. Rg3 increases the expression of lncRNA TUG1 and reduces the expression of miR-200a-3p to stimulate the SIRT1/AMPK pathway, thus improving liver injury. SIRT1 is involved in the development of liver fibrosis through the regulation of lncRNA MALAT1.

SIRT1 is involved in the apoptosis and reversal of activated stellate cells via the regulation of lncRNA MALAT1, thereby preventing liver fibrosis (Figure 5) [80]. Meanwhile, drugs or plant extracts can participate in regulating the abnormal expression of lncRNAs, which may help alleviate inflammatory diseases. Gu et al. [78] found that dexmedetomidine hydrochloride (DEX) significantly elevated the expression of lncRNA TUG1 in oxygen and glucose deprivation (OGD)-induced WRL-68 cells. The overexpressed lncRNA TUG1 can suppress the inflammatory response of hepatocytes in liver injury via miR-194/SIRT1 axis (Figure 5). In addition, ginsenoside Rg3 increased the expression of lncRNA TUG1, and the overexpressed lncRNA TUG1 subsequently activated the SIRT1/AMPK pathway by targeting miR-200a-3p, thus improving liver injury (Figure 5) [79]. In summary, lncRNAs are involved in regulating the development of liver inflammation. In liver inflammation, the strategy of regulating lncRNA expression has been successfully implemented in preclinical models. However, the safety and reliability of lncRNAs in human application still face great challenges.

### 4.3. Role of lncRNAs in Inflammatory Lung Injury

ALI, one of the inflammatory diseases of the lung, has been studied extensively in recent years. ALI is a multifactorial disease directly related to conditions such as pneumonia and pulmonary contusion and closely related to sepsis, endotoxin infection, and others. An LPS-induced lung injury model has been widely used in the study of lung injury [119]. In recent years, researchers have found that some lncRNAs, including lncRNA MALAT1 [120], lncRNA HOTAIR [121], and lncRNA NLRP3 [122], play a key role in regulating inflammatory lung diseases.

**Figure 6:**
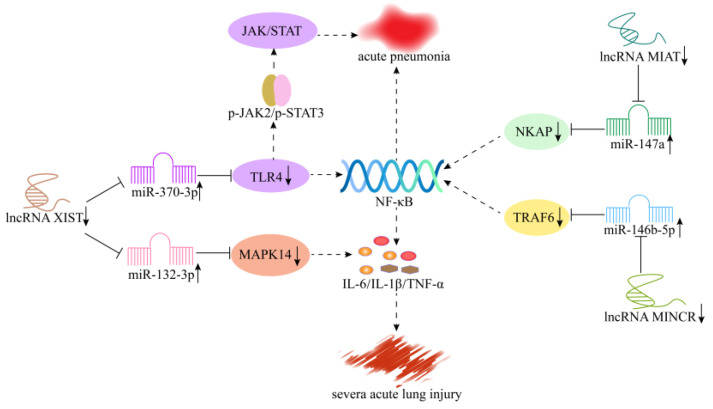
Mechanism of lncRNAs in lung inflammatory injury. Knockdown of lncRNA XIST regulates the JAK/STAT3 and NF-κB signaling pathways by targeting miR-370-3p/TLR4, thereby inhibiting acute pneumonia. Knockdown of lncRNA XIST alleviates acute lung injury by regulating the miR-132-3p/MAPK14 pathway. Downregulation of lncRNA MINCR regulates the TRAF6 expression levels by targeting miR-146b-5p, thereby inhibiting NF-κB activation and attenuating ALI. Low expression of lncRNA MIAT inhibits the activation of NF-κB pathway by targeting miR-147a/NKAP axis, thereby alleviating the damage of pneumonia.

LncRNA XIST was discovered in the 1990s [123,124]. Increasingly, studies reveal that lncRNA XIST dysregulation plays an important role in the pathological process of many diseases, such as coronary artery disease [125], renal fibrosis [126], and myocardial injury [127]. Moreover, lncRNA XIST significantly increased in the serum from patients with acute pneumonia and in LPS-induced human lung fibroblasts of WI-38, playing a regulatory role as a ceRNA. Zhang et al. [81] found that knockdown of lncRNA XIST inhibited the expression of TLR4 by targeting miR-370-3p, thereby regulating the JAK/STAT3 and NF-κB signaling pathways, inhibiting cellular apoptosis and the secretion level of inflammatory cytokines, and reducing LPS-induced cell damage; this process may be used to develop a strategy for treating acute pneumonia (Figure 6). Li et al. [82] showed that knockdown of lncRNA XIST alleviated cell death and LPS-induced lung injury by regulating the miR-132-3p/MAPK14 pathway (Figure 6).

In LPS-induced ALI, Gao et al. [83] found that the expression of lncRNA MINCR was significantly upregulated, while downregulation of lncRNA MINCR regulated the TRAF6 expression levels by targeting miR-146b-5p, thereby inhibiting NF-κB activation and inflammatory factor secretion and, ultimately, attenuating ALI (Figure 6). The low expression of lncRNA MIAT reduces NKAP expression by targeting miR-147a, inhibits NF-κB pathway activation, and alleviates the damage of LPS-induced pneumonia (Figure 6) [84]. According to Zhou et al. [85], the downregulation of lncRNA NEAT1 inhibits the HMGB1/RAGE-NF-κB signaling pathway, protecting against LPS-induced alveolar epithelial cell (AECs) injury and inflammation. In addition, in a sepsis-induced lung injury model, Qiu et al. [86] detected a low expression of lncRNA TUG1, and overexpression of lncRNA TUG1 ameliorated sepsis-induced lung injury, secretion of proinflammatory cytokines, and apoptosis by suppressing miR-34b-5p and promoting GRB2-associated binding protein 1 (GAB1). Therefore, lncRNA TUG1 may be used as a potential therapeutic target for sepsis-induced ALI.

At present, auxiliary ventilation and drug therapy are the main therapeutic methods for ALI. However, specific therapeutic targets are still lacking. LncRNAs play important roles in the pathogenesis of ALI, so they may become new diagnostic and therapeutic markers for ALI. Therefore, the regulatory mechanisms and signaling pathways involved in the pathogenesis of ALI need to be elucidated to provide targeted drugs for clinical treatment. The abovementioned differentially expressed lncRNAs regulate the occurrence of inflammatory response in ALI by binding with miRNAs, which may be a potential therapeutic target and a basis for ALI drug therapy.

### 4.4. Role of lncRNAs in OA

OA is a progressive joint disease and one of the most common types of arthritis. Cartilage, subchondral bone, and synovium may all play a key role in the pathogenesis of the disease [128]. Chondrocyte degeneration is an important factor in cartilage destruction, and the proper regulation of chondrocyte proliferation, apoptosis, autophagy, and secretion is the key to prevent and treat OA. It has been reported that lncRNAs participate in the occurrence and development of OA, and their abnormal expression may lead to change in cellular behavior [129,130].

**Figure 7:**
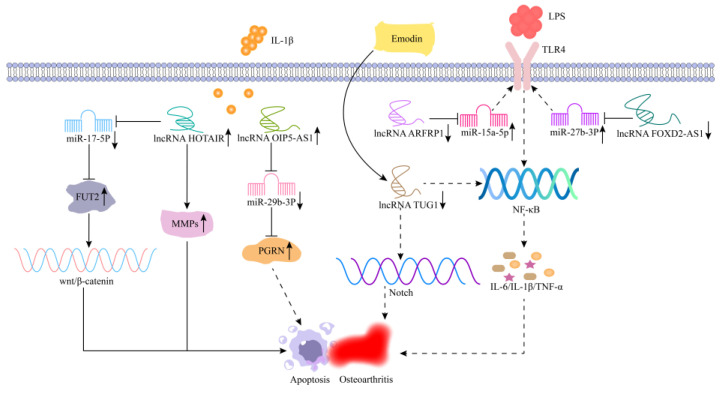
Mechanism of lncRNAs in osteoarthritis. In IL-1β-induced OA model, overexpression of lncRNA HOTAIR promotes the expression of MMPs and exacerbates OA. Overexpression of lncRNA HOTAIR increases the activity of Wnt/β-catenin pathway by regulating miR-17-5p/FUT2 axis, which aggravates the injury and apoptosis of chondrocytes. Overexpression of lncRNA OIP5-ASI inhibits apoptosis and inflammation by targeting miR-29b-3p/PGRN. Emodin inhibits the Notch/NF-κB pathway by upregulating lncRNA TUG1, which alleviates the apoptotic and inflammatory response of chondrocytes. LncRNA ARFRP1 is significantly upregulated in LPS-induced chondrocytes, and the downregulation of lncRNA ARFRP1 inhibits activation of NF-κB signaling pathway by targeting miR-15a-5p/TLR4 axis. Knockdown of lncRNA FOXD2-AS1 alleviates inflammatory injury by regulating the miR-27a-3p/TLR4 axis.

IL-1β is produced by various cells, including macrophages, chondrocytes, and synoviocytes, and plays a crucial role in the development of OA [131]. The IL-1β-induced OA model can further validate the function of related lncRNAs in the occurrence of OA. Zhang et al. [87] revealed that lncRNA HOTAIR expression was significantly upregulated in an IL-1β-induced OA model, which promoted the expression of matrix metalloproteinases (MMPs) and exacerbated the inflammatory damage in chondrocytes (Figure 7). In addition, in OA cartilage tissue, the overexpression of lncRNA HOTAIR sponged miR-17-5p to regulate the expression of fucosyl transferase (FUT2) in chondrocytes and increased the activity of the Wnt/β-catenin pathway, which aggravated the injury and apoptosis of chondrocytes (Figure 7). These results provide a new target for the molecular therapy of OA [88]. LncRNA OIP5-AS1 is a newly discovered lncRNA, and Zhi et al. [89] found that the expression of lncRNA OIP5-ASI was significantly downregulated in an IL-1β-induced OA model. The overexpression of lncRNA OIP5-ASI promoted chondrocyte viability and migration and inhibited apoptosis and inflammation by miR-29b-3p/PGRN (Figure 7).

In the LPS-induced OA model, emodin can inhibit the Notch/NF-κB pathway by upregulating lncRNA TUG1 and alleviate the apoptotic and inflammatory response of chondrocytes (Figure 7) [90]. According to Zhang et al. [91], lncRNA ARFRP1 was significantly upregulated in OA cartilage tissue and LPS-induced chondrocytes. The downregulation of lncRNA ARFRP1 inhibited activation of NF-κB signaling pathway by targeting miR-15a-5p/TLR4 axis [91]. This mechanism improves inflammatory damage in OA chondrocytes and tissues (Figure 7). In addition, lncRNA FOXD2-AS1 increased the mRNA and protein levels of TLR4 through the sponge action of miR-27a-3p, promoting the inflammatory response in OA [92]. Thus, knockdown of lncRNA FOXD2-AS1 can alleviate inflammatory injury by regulating the miR-27a-3p/TLR4 axis (Figure 7). Li et al. [93] showed that the silencing of lncRNA MIAT could protect ATDC5 chondrocytes from LPS-induced damage, by targeting miR-132 to inhibit the NF-κB and JNK signaling pathways.

In summary, many lncRNAs are differentially expressed in human OA chondrocytes, and a few lncRNAs have been confirmed to participate in the inflammatory reaction process associated with OA chondrocytes, suggesting that inhibiting or overexpressing key lncRNAs can help treat or alleviate OA. However, most studies on the role of lncRNAs in OA have used only synovial tissues from patients with OA, and the sample size is relatively small in these studies. Therefore, a larger sample of patients with OA is needed to further validate the function of key lncRNAs in OA.

### 4.5. Role of lncRNAs in Mastitis

Mastitis is mainly caused by the invasion of pathogenic bacteria in the mammary gland and has complex pathogenesis. The main pathogens that cause mastitis are *Staphylococcus aureus*, *Streptococcus uberis*, and *Escherichia coli* [132,133]. Compared with advances in the study of lncRNAs in the regulation of inflammatory diseases in humans and mice, the study of lncRNAs in the regulation mechanism of mastitis has been slow. Mumtaz et al. [134] identified 112 differentially expressed lncRNAs in goat mammary epithelial cells induced by *E. coli* and *S. aureus*. In addition, Wang et al. [135] also found 112 differentially expressed lncRNAs in LPS-treated bovine mammary epithelial cells, and these lncRNAs participated in inflammation-related signal pathways (i.e., the Notch, NF-κB, MAPK, and PI3K-AKT signal pathways). A large amount of the lncRNA information in the present study may provide clues for functional and molecular studies of mammary epithelial cells–bacteria interaction.

**Figure 8:**
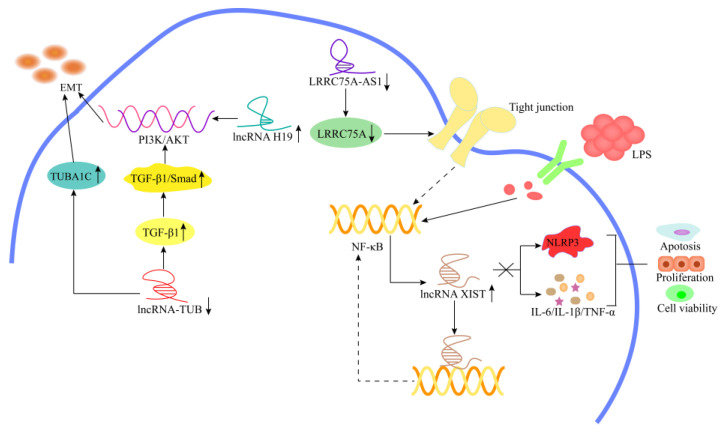
Mechanism of lncRNAs in the modulation of mastitis. Overexpression of lncRNA H19 promotes TGF-β1-induced EMT via PI3K/AKT signaling pathway. Knockdown of lncRNA-TUB results in the downregulation of TUBA1C and the upregulation of TGF-β1, and the increased secretion of TGF-β1 activates the TGF-β1/Smad pathway, ultimately promoting EMT. LncRNA XIST mediates cell proliferation, viability, and apoptosis via generating a negative feedback regulation of NF-κB/NLRP3 inflammasome pathway. Downregulated LRRC75A-AS1 inhibits the NF-κB pathway by enhancing the expression of TJ protein, thereby alleviating the inflammatory response of MAC-T cells.

In bovine mastitis, research has been primarily concentrated on a few lncRNAs, including lncRNA H19, LRRC75A-AS1, lncRNA TUB, and lncRNA XIST. Yang et al. [94] showed that lncRNA H19 expression was significantly increased in both LPS- and lipoteichoic acid (LTA)-induced inflammatory MAC-T cells, and the overexpression of lncRNA H19 promoted TGF-β1-induced EMT and the overaccumulation of extracellular matrix (ECM) proteins, which led to the formation of breast fibrosis (Figure 8). Li et al. [5] reported that overexpressed lncRNA H19 promoted the activation of the NF-κB pathway, which resulted in the timely clearance of bacteria and toxic substances, and enhanced the immune response of MAC-T cells. At the same time, the overexpression of lncRNA H19 can enhance the expression of MAC-T cell β-casein tight-junction-related proteins (claudin-1, occludin, and ZO-1) and restores the blood–milk barrier, which is important for the recovery of breast function after infection. Bovine mastitis usually causes a series of pathological changes in the body, with lncRNA TUB affecting EMT in the bovine mammary epithelial cells (bMECs) of cows. Wang et al. [95] identified 1323 lncRNAs in MAC-T cells via bioinformatics; of these, 53 were differentially expressed, and most of the 53 were involved in the pathogenesis of bovine mastitis. To further evaluate the functions of the predicted mastitis-associated lncRNAs, a novel lncRNA TUB with significantly upregulated expression was identified in inflammatory MAC-T. Knockdown of lncRNA TUB significantly reduced the expression of TUBA1C, leading to EMT and inhibiting cell proliferation, migration, and β-casein secretion. In addition, lncRNA TUB knockdown promoted the secretion of TGF-β1 and activated the TGF-β1/Smad pathway to participate in EMT (Figure 8) [95]. Ma et al. [96] found that *S. aureus* and *E. coli*-induced bMECs rapidly activated the NF-κB signaling pathway, resulting in the upregulation of lncRNA XIST expression. However, the highly expressed lncRNA XIST negatively inhibited the NF-κB pathway, which inhibited the formation of NLRP3 and the secretion of inflammatory cytokines, alleviating the inflammatory response of mammary epithelial cells in cows (Figure 8). LRRC75A-AS1 is an approximately 4 kb lncRNA transcribed from the antisense strand of the LRRC75A gene. LRRC75A-AS1 was significantly decreased in *E. coli*-induced MAC-T cells. Moreover, the downregulated LRRC75A-AS1 inhibited the NF-κB pathway by enhancing the expression of TJ protein, thereby alleviating the inflammatory response of MAC-T cells (Figure 8) [3]. Zhang et al. [97] found that lncRNA NONBTAT017009.2 interacted with miR-21-3p to upregulate IGFBP5 expression, which subsequently decreased the viability and proliferation of bMECs and reduced lactation performance in cows. Yang et al. [98] showed that lncRNA TCONS_00015196 and lncRNA TCONS_00087966 improved the proliferation and viability of bMECs by targeting miR-221. Meanwhile, lncRNA MPNCR inhibited the proliferation of bMECs via the miR-31/CAMK2D axis [45].

The pathogenesis and progression of mastitis are extremely complex processes. It has been confirmed that lncRNAs are closely associated with mastitis, but the molecular mechanism of lncRNAs in regulating mastitis is yet unclear. Only a few differentially expressed lncRNAs have been identified in mastitis, so more lncRNAs need to be evaluated to provide new ideas for the diagnosis and treatment of mastitis.

### 4.6. Role of lncRNAs in Central Nervous System Inflammation

**Figure 9:**
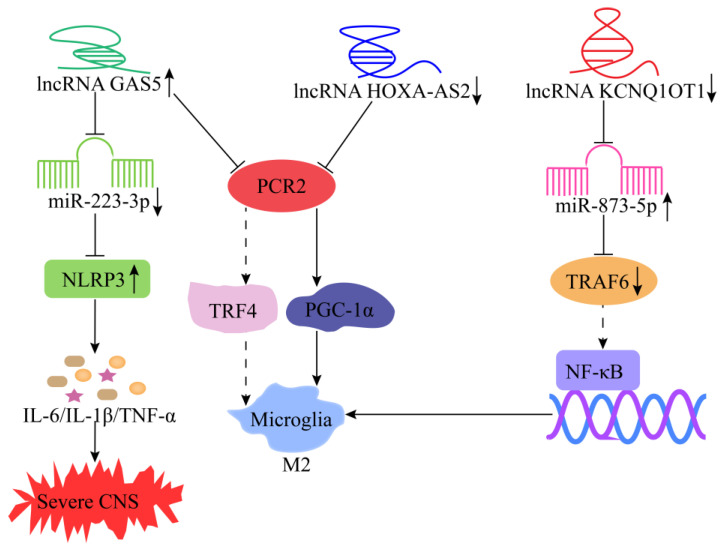
Mechanism of lncRNAs in the modulation of CNS inflammation. Knockdown of lncRNA KCNQ1OT1 promotes M2 polarization in microglia by targeting the miR-873-5p/TRAF6 axis. LncRNA GAS5 represses TRF4 transcription by binding to PCR2, thereby inhibiting microglia M2 polarization. Knockdown of lncRNA HOXA-AS2 increases PGC-1α expression by binding to PCR2, which, consequently, promotes microglia M2 polarization. LncRNA GAS5 suppresses transcription of TRF4 by recruiting the PRC2, thus inhibiting M2 polarization. LncRNA GAS5 accelerates PD progression by targeting miR-223-3p/NLRP3 axis.

Inflammation in the central nervous system (CNS) mainly includes traumatic brain injury (TBI), multiple sclerosis (MS), and neurodegenerative diseases such as Alzheimer’s disease (AD) and Parkinson’s disease (PD) [136,137]. Microglia, the resident macrophages of the CNS, play an important physiological function in CNS inflammation and maintaining tissue homeostasis [138,139]. Several studies have shown that lncRNAs can regulate M1/M2 polarization of microglia and act as a biomarker of CNS inflammation [140,141,142,143]. Moreover, scholars recently revealed that many lncRNAs participate in regulating the processes of CNS inflammation (Table 3, Figure 9), which will lay a foundation for the development of molecular therapy strategies for CNS inflammation.

## 5. Conclusions and Future Perspectives

LncRNAs regulate the expression of coding genes by binding to miRNA, mRNA, DNA, or proteins. LncRNAs regulate inflammatory responses as well as the proliferation, differentiation, and polarization of many immune cells. At the physiological level, they regulate renal inflammation, hepatic inflammation, pneumonia, OA, mastitis, and central system inflammation. Therefore, elucidating the molecular mechanism of lncRNAs in immune regulation can provide novel strategies for the development of early diagnostics and molecular therapy for inflammatory diseases. In humans and animals, although many lncRNAs have been identified and have been shown to be potential molecular markers for the diagnosis and prognosis of several diseases, most available evidence is derived from in vitro or cell line studies, and the specificity and sensitivity of these lncRNAs are still insufficient for clinical application. In addition, conservation among species limits the validation of lncRNAs functions in vivo, and further large-scale prospective studies are necessary. In addition, a large number of differentially expressed lncRNAs are associated with cellular or systemic inflammation. The functions of the more effective lncRNAs associated with inflammatory diseases should be explored to supplement the involved molecular network and provide avenues for developing clinical molecular therapy for inflammatory diseases.

## Figures and Tables

**Table 1:** Function of intracellular long noncoding RNAs.

Cell Types	lncRNA	Target	Function	References
Macrophages	lncRNA Mirt2 lncRNA NEAT1 lncRNA NEAT1 lncRNA IGHCγ lncRNA HIX003209 lnc MC	— miR-125a-5p — miR-6891-3p miR-6989 miR-199a-5p	Regulate macrophage differentiation, polarization, proinflammatory cytokine release, and inflammatory injury	[16] [17] [18] [19] [20] [21]
Dendritic cells	lncRNA Dpf3 lncRNA NEAT1 lncRNA MALAT1 lncRNA MALAT1 lnc-DC lnc-DC	HIF-1α miR-3076-3p miR-155 miR-155-5p STAT3 —	Regulate the migration, maturation, differentiation, and inflammatory injury of dendritic cells	[22] [23] [24] [25] [26] [27]
T cells	linc-MAF-4 lncRNA AW112010 lncRNA IFNG-AS1 lncRNA GAS5	— KDM5A — miR-92a-3p	Regulate T cell differentiation	[28] [29] [30] [31]
Endothelial cells	lncRNA H19 lncRNA OIP5-AS1 lncRNA MALAT1 lncRNA MEG3 lncRNA SNHG12 lncRNA HOTAIR	miR-let-7 miR-98-5p miR-590 miR-223 miR-25-3p miR-22	Attenuate endothelial cell injury	[32] [33] [34] [35] [36] [37]
Epithelial cells	lncRNA H19 lncRNA MEG3 lncRNA 105377478 lncRNA Hsp4 lncRNA NEAT1 lncRNA NEAT1 lncRNA TUG1 lncRNA MPNCR	miR-19b miR-34a AdipoR1 miR-466m-3p miR-582-5p miR-93-5p miR-223 miR-31	Attenuate epithelial cell injury	[38] [39] [40] [41] [42] [43] [44] [45]

**Table 2:** Function of long noncoding RNAs in various inflammatory diseases.

Inflammatory Disease	lncRNA	Target	Function	References
Acute kidney injury (AKI)	lncRNA CCAT1 lncRNA GRNDE lncRNA MALAT1 lncRNA MALAT1 lncRNA PVT1	miR-155 miR-181a-5p miR-370-3p miR-146a —	Alleviate AKI	[70] [71] [72] [73] [74]
Hepatic inflammation	lncRNA HOTAIR lncRNA XIST lncRNA TUG1 lncRNA TUG1 lncRNA TUG1 lncRNA MALAT1	— BRD4 miR-140 miR-194 miR-200a-3p —	Alleviate hepatic inflammation	[75] [76] [77] [78] [79] [80]
Acute lung injury (ALI)	lncRNA XIST lncRNA XIST lncRNA MINCR lncRNA MIAT lncRNA NEAT1 lncRNA TUG1	miR-370-3p miR-132-3p miR-146b-5p miR-147a — miR-34b-5p	Alleviate ALI	[81] [82] [83] [84] [85] [86]
Osteoarthritis (OA)	lncRNA HOTAIR lncRNA HOTAIR lncRNA OIP5-ASI lncRNA TUG1 lncRNA ARFRP1 lncRNA FOXD2-AS1 lncRNA MIAT	— miR-17-5p miR-29b-3p — miR-15a-5p miR-27a-3p miR-132	Alleviate OA	[87] [88] [89] [90] [91] [92] [93]
Mastitis	lncRNA H19 lncRNA TUB lncRNA XIST LRRC75A-AS1 NONBTAT017009.2 TCONS_00015196 TCONS_00087966 lncRNA MPNCR	— TUBA1C — LRRC75A miR-21-3p miR-221 miR-221 miR-31	Alleviate mastitis	[5,94] [95] [96] [3] [97] [98] [98] [45]
Central nervous system inflammation	lncRNA MEG3 lncRNA MALAT1 lncRNA MALAT1 lncRNA KCNQ1OT1 lncRNA GAS5 lncRNA GAS5 lncRNA Gm13568 lncRNA DDIT4 lncRNA H19 lncRNA H19 lncRNA ATB lncRNA HOXA-AS2	miR-7a-5p — miR-129 miR-873-5p PCR2 miR-223-3p — DDIT4 miR-129 miR-585-3p miR-200 PCR2	Alleviate central nervous system inflammation	[99] [100] [101] [102] [103] [104] [105] [106] [107] [108] [109] [110]

**Table 3:** The role of lncRNAs in the regulation of central nervous system inflammation.

lncRNA	CNS Inflammation Type	Expression	Molecular Mechanism	References
lncRNA MEG3	TBI	Upregulated	LncRNA MEG3 regulates microglia activation and inflammatory response by targeting the miR-7a-5p/NLRP3 axis.	[99]
lncRNA MALAT1	TBI	Downregulated	Overexpression of lncRNA MALAT1 reduces the expression of IL-6, NF-κB, and AQP4, thereby alleviating TBI-induced inflammatory injury.	[100]
lncRNA KCNQ1OT1	TBI	Upregulated	Knockdown of lncRNA KCNQ1OT1 can promote M2 polarization in microglia by targeting the miR-873-5p/TRAF6 axis, thereby alleviating the TBI-mediated inflammatory response (Figure 9).	[102]
lncRNA GAS5	MS	Upregulated	LncRNA GAS5 represses TRF4 transcription by binding to PCR2, thereby inhibiting microglia M2 polarization and, ultimately, exacerbating the progression of MS (Figure 9).	[103]
lncRNA DDIT4	MS	Upregulated	Overexpression of lncRNA DDIT4 alleviates the development of MS by inhibiting the DDIT4/mTOR axis.	[106]
lncRNA Gm13568	MS	Upregulated	Inhibition of lncRNA Gm13568 attenuates the activation of Notch signal pathway, thereby alleviating demyelination in EAE mice.	[105]
lncRNA H19	AD	Upregulated	LncRNA H19 inhibits AD cell apoptosis and oxidative stress by targeting the miR-129/HMGB1 axis.	[107]
lncRNA ATB	AD	Upregulated	Inhibition of lncRNA ATB alleviates development of AD by targeting miR-200 to inhibit the expression of HMGB1.	[109]
lncRNA H19	PD	Downregulated	Overexpression of lncRNA H19 upregulates PIK3R3 expression by targeting miR-585-3p, thereby attenuating MTPT-induced neuronal apoptosis.	[108]
lncRNA HOXA-AS2	PD	Upregulated	Knockdown of lncRNA HOXA-AS2 can increase PGC-1α expression by binding to PCR2, thereby promoting microglia M2 polarization and ultimately alleviating the development of PD (Figure 9).	[110]
lncRNA MALAT1	PD	Upregulated	Resveratrol inhibits the expression of lncRNA MALAT1 in PD mice, and the low expression of lncRNA MALAT1 can reduce the expression of SNCA by targeting miR-129, thereby inhibiting neuronal apoptosis and alleviating PD.	[101]
lncRNA GAS5	PD	Upregulated	LncRNA GAS5 exacerbates PD development by targeting and regulating the miR-223-3p/NLRP3 axis (Figure 9).	[104]

## Data Availability

Not applicable.
